# Continuous venovenous hemodiafiltration in the treatment of newborns with an inborn metabolic disease: a single center experience

**DOI:** 10.3906/sag-1811-8

**Published:** 2020-02-13

**Authors:** Hasan AKDUMAN, Emel OKULU, Fatma Tuba EMİNOĞLU, Tanıl KENDİRLİ, Gaffari TUNÇ, Ebru AZAPAĞASI, Oktay PERK, Ömer EREDEVE, Begüm ATASAY, Saadet ARSAN

**Affiliations:** 1 Department of Neonatology, University of Health Sciences, Dr. Sami Ulus Maternity and Children Research and Training Hospital, Ankara Turkey; 2 Department of Pediatrics, Division of Neonatology, Faculty of Medicine, Ankara University, Ankara Turkey; 3 Department of Pediatrics, Division of Pediatric Metabolic Diseases, Faculty of Medicine, Ankara University, Ankara Turkey; 4 Department of Pediatrics, Division of Pediatric Intensive Care Unit, Faculty of Medicine, Ankara University, Ankara Turkey

**Keywords:** Inborn metabolic diseases, renal replacement therapy, continuous venovenous hemodiafiltration, newborn, survival

## Abstract

**Background/aim:**

Most inborn metabolic diseases are diagnosed during the neonatal period. The accumulation of toxic metabolites may cause acute metabolic crisis with long-term neurological dysfunction and death. Renal replacement therapy (RRT) modalities allow the efficient removal of toxic metabolites. In this study, we reviewed our experience with continuous venovenous hemodiafiltration (CVVHDF) as RRT for newborns with an inborn metabolic disease.

**Materials and methods:**

Patients diagnosed with an inborn metabolic disease and who received CVVHDF treatment at our neonatal intensive care unit between January 2014 and December 2017 were included in this study. Their demographic and clinical data were collected, and the efficacy and safety of CVVHDF was evaluated.

**Results:**

A total of nine continuous RRT (CRRT) sessions as CVVHDF were performed in eight newborns with a diagnosis of urea cycle defect (n = 5), maple syrup urine disease (n = 2), or methylmalonic acidemia (n = 1). The mean age at admission was 10 ± 8.6 days (range: 3–28 days). The mean plasma levels of ammonium were 1120 ± 512.6 mg/dL and 227.5 ± 141.6 mg/dL before and at the end of the treatment, respectively. Plasma levels of leucine were 2053.5 ± 1282 µmol/L and 473.5 ± 7.8 µmol/L before and at the end of the treatment, respectively. The CVVHDF duration was 32.3 ± 11.1 h (median: 37 h; range: 16–44 h), and the mean length of hospitalization was 14.6 ± 12.9 days. The mean duration of CVVHDF was 32.3 ± 11.1 h (range: 16–44 h). Circuit clotting was the most common observed complication (37.5%) and the survival rate was 50%. Among surviving patients, two developed severe and two developed mild mental and motor retardation.

**Conclusion:**

CVVHDF is a CRRT modality that can be used to treat newborns with an inborn metabolic disease. Early diagnosis, commencement of specific medical therapy, diet, and extracorporeal support, if needed, are likely to result in improved short and long-term outcomes.

## 1. Introduction

Newborns with an inborn metabolic disease often present with coma and require urgent diagnosis, nutritional support with specific medical therapy, and toxin removal [1]. Most inborn metabolic diseases are diagnosed during the neonatal period. The time to diagnosis is critical in preventing neurological and developmental dysfunction due to the accumulation of toxic metabolites [2–4]. Renal replacement therapy (RRT) modalities allow for efficient removal of toxic metabolites, and minimize the duration of exposure to these metabolites while diagnostic studies are being performed [5–8]. 

Infants in metabolic crisis requiring extracorporeal therapy are managed with various modalities favorable for the rapid clearance of toxins. The currently available RRT modalities consist of peritoneal dialysis (PD), intermittent hemodialysis (HD), and forms of continuous extracorporeal removal including continuous venovenous hemofiltration (CVVH), continuous venovenous hemodialysis (CVVHD), and continuous venovenous hemodiafiltration (CVVHDF) [9,10]. Donn et al. concluded that HD is the preferred modality for treating hyperammonemia caused by a urea cycle defect (UCD) compared to exchange transfusion and PD [11]. In recent years, HD, particularly continuous techniques, has become suitable for newborns following its increasing application and technical developments [12–14].

In this report, we present our single-center experience using CVVHDF to treat newborns with an inborn metabolic disease.

## 2. Materials and methods

### 2.1. Patients

Patients diagnosed with an inborn metabolic disease who received CVVHDF treatment at our neonatal intensive care unit between January 2014 and December 2017 were enrolled in this study. Their demographic and clinical data were collected retrospectively. The study was approved by the local ethics committee.

The diagnoses were as follows: UCD in five patients, maple syrup urine disease (MSUD) in two patients, and methylmalonic acidemia (MMA) in one patient. The efficacy and safety of the chosen method for removing toxic metabolites were evaluated. 

### 2.2. Medical therapy 

General supportive care including respiratory and circulatory support and correction of electrolyte imbalances was provided to all patients. Patients received specific treatments according to their diagnosis, as detailed below [15,16]: 

**UCD:** A high caloric intake and protein-free parenteral nutrition was provided. Intravenous sodium phenyl acetate and sodium benzoate (Ammonul) at a loading dose of 250 mg/kg, followed by a maintenance infusion of 250–300 mg/kg/day, was started. In addition, L-arginine hydrochloride was administered at a starting dose of 2 mmoL/kg, followed by a continuous infusion of 2 mmoL/kg/day. L-carnitine was administered at a dose of 100 mg/kg/day. 

**MSUD:** Hypercaloric parenteral nutrition (110–130 kcaL/kg/day) and thiamine supplementation (10 mg/kg/day) were administered. To achieve an anabolic state and provide the total caloric intake, we administered high-concentration dextrose containing fluids and intravenous lipids intravenously (2–3 g/kg/day). As parenteral branched-chain amino acid (BCAA)-free amino acid solutions were unavailable in our country, amino acids were not used in our parenteral nutrition regimen.

**MMA:** Intravenously administered high-concentration dextrose containing fluids and intravenous lipids (2–3 g/kg/day) was given to provide the total caloric intake with low or free protein. L-carnitine was given at a dose of 100 mg/kg/day. Cobalamin (vitamin B12) was administered as an injection. 

The patients, who had ammonium level > 500 µmol/L or leucine level > 1200 µmol/L or had metabolic acidosis that is unresponsive to medications or had signs of encephalopathy, immediately decided to receive RRT. 

### 2.3. Vascular access and dialysis 

A pediatric intensivist inserted a dual lumen catheter into an internal jugular vein or femoral vein using the Seldinger technique. The length of the catheter was adjusted according to the patient’s weight [17]. Hemodiafiltration was introduced with a Prismaflex (Baxter, USA) device, and HF20 and M60 hemofilters were used (Baxter, USA). The patient’s body weight was also used to select the appropriate filter. Whole blood product was used to prime the extracorporeal blood circuit.

Blood flow and dialysate flow velocities were chosen according to the weight and blood pressure of the patient (blood flow: 8–12 mL/kg/min, dialysate flow: 2000 mL/1.73 m2 per hour). Anticoagulation was achieved with unfractionated heparin with a loading dose of 50 IU/kg and followed by continuous infusion at a dose of 10 IU/kg per hour. The dosage of heparin was adjusted according to the activated partial thromboplastin time, which was checked every 6 h. 

### 2.4. Statistical analyses

Data were analyzed using the SPSS 12 (IBM Corp., Armonk, NY, USA) for Windows. Mean ± standard deviation, median, and percentages are presented. Paired t-test is used to compare the means.

## 3. Results

A total of nine CRRT sessions were performed in eight newborns during the study period. The indications for CRRT were an inborn metabolic disease, as follows: UCD in five patients, MSUD in two patients, and MMA in one patient. 

The patients included five boys (62.5%) and three girls (37.5%). The mean gestational age and mean body weight of the patients were 39.3 ± 0.8 weeks (median: 39.2 weeks, range: 38–40.5 weeks) and 3388 ± 549 g (median: 3250 g, range: 2800–4400 g), respectively. The median age at admission was 10 ± 8.6 days (median: 7 days, range: 3–28 days). Poor feeding and respiratory failure were the most relevant signs on admission (Table 1). The catheter sizes used were 7 Fr in five (62.5%) patients, 5 Fr in two (25%) patients, and 8 Fr in one (12.5%) patient. A total of six (75%) catheters were inserted into the internal jugular vein and two (25%) into the femoral vein. Five patients (62.5%) had received inotropes prior to CVVHDF.

**Table 1:** The demographic and clinical findings of patients on admission.

No.	Gestationalage (wk)	Sex	Age onadmission (d)	Weight on admission (g)	Symptoms on admission	Diagnosis	Duration of hospitalization (d)	Outcome	Current age (months)
1	40.5	M	9	3400	Poor feeding, vomitting	UCD	15	Discharged	36
2	39	F	3	2900	Poor feeding, respiratory failure	UCD	4	Died	-
3	40	F	3	3000	Seizure, respiratory failure	UCD	41	Discharged	45
4	40	M	4	3100	Poor feeding, respiratory failure	UCD	8	Died	-
5	39	M	15	2800	Poor feeding, vomiting, respiratory failure	UCD	4	Died	-
6	38	F	5	3700	Poor feeding, vomitting	MMA	4	Died	-
7	39.5	M	28	4400	Seizure, respiratory failure	MSUD	24	Discharged	48
8	39	M	13	3800	Poor feeding, seizure	MSUD	17	Discharged	50

F: female, M: male, MSUD: maple syrup urine disease, MMA: methyl malonic academia, UCD: urea cycle defect.

The mean blood flow rate was 34.4 ± 8.2 mL/kg/min (median: 30 mL/kg/min, range: 25–50 mL/kg/min) and the mean dialysate rate was 2150 ± 0.13 mL/1.73 m2 per hour (median: 2200 mL/1.73 m2 per hour, range: 2000–2300 mL/1.73 m2 per hour). The mean plasma level of ammonium was reduced from 1120 ± 512.6 mg/dL [median: 1097 µmol/L, range: 451–1728 µmol/L, (normal range: 16-53 µmol/L)] to 227.5 ± 141.6 mg/dL (median: 190.5 mg/dL, range: 96–484 µmol/L) at the end of the treatment compared to the level before the initiation of CVVHDF (P = 0.007). The mean reduction rate of ammonium was 76.5 ± 16.2% per hour (median: 84.7% per hour, range: 53%–89.6% per hour) and 14.7 ± 15.5% per hour (median: 8.7% per hour, range: 3.5%–44.8% per hour), before and after treatment, respectively. The mean plasma levels of leucines were 2053.5 ± 1282 µmol/L [range: 1147–2960 µmol/L, (normal range: 55-149 µmol/L)] and 473.5 ± 7.8 µmol/L (range: 468–479 µmol/L) (P = 0.33), and the mean reduction rate of leucine was 71.5 ± 17.4% per hour (range: 59.2%–83.8% per hour) and 5.6 ± 2% per hour (range: 4.2%–7% per hour), respectively (Table 2).

**Table 2:** The treatment data of the patients.

No.	Dialysis modality	Vascular access	Catheter size	Blood flowrate (mL/min)	Dialysate rate(mL/1.73 m2per hour)	Initial plasma ammonia level (µmol/L)	Control plasma ammonia level (µmol/L)	Ammonia reduction rate %	Time to reduction (h)	Reductionrate % per h	CRRT duration (h)
1	CVVHDF	V. femoralis	8 Fr	40	2000	1164	121	89.6	2	44.8	22
2	CVVHDF	V. jugularis interna	5 Fr	30	2200	1663	196	88.2	9	9.8	44
3	CVVHDF	V. jugularis interna	7 Fr	30	2000	1728	283	83.6	11	7.6	42
4	CVVHDF	V. jugularis interna	7 Fr	30	2300	682	96	85.9	5	17.2	36
5	CVVHDF	V. femoralis	7 Fr	30	2300	1030	484	53	15	3.5	38
6	CVVHDF	V. jugularis interna	7 Fr	25	2000	451	185	59	11	5.4	40
						Initial plasma leucine level (µmol/L)	Control plasma leucine level (µmol/L)	Leucine reduction rate %	Time to reduction (h)	Reductionrate % per h	
7	CVVHDF	V. jugularis interna	5 Fr	50	2200	1147	468	59.2	14	4.2	20
8	CVVHDF	V. jugularis interna	7 Fr	40	2200	2960	479	83.8	12	7	16

CVVHDF; continuous venovenous hemodiafiltration.

The mean CVVHDF duration was 32.3 ± 11.1 h (median: 37 h, range: 16–44 h). Circuit clotting was observed in three courses (37.5%) of CVVHDF, whereas bleeding complications were observed in one patient (patient 4, 12.5%) with UCD, which was resolved by ceasing heparin. No infections were observed during the study period. A total of five of the eight patients (62.5%) developed thrombocytopenia at the end of 24 h of CRRT, which resolved without the need for any intervention.

The mean length of hospitalization was 14.6 ± 12.9 days (median: 11.5 days, range: 4–41 days) and the survival rate was 50%. Among those who survived, two patients (patients 1 and 2) developed severe, and a further two patients (patients 7 and 8) developed mild mental and motor retardation.

## 4. Discussion 

The accumulation of toxic metabolites in infants with an inborn metabolic disease may cause acute metabolic crisis and result in long-term neurological dysfunction and death. Lowering the concentration of these metabolites can prevent neurological damage and may be lifesaving [7]. RRTs have been successfully used to reduce the plasma levels of small-molecular-weight solutes [12]. In a retrospective analysis of our patients with an inborn metabolic disease, CVVHDF was used effectively and provided a fast reduction in toxic metabolites. 

CRRT is becoming increasingly popular in pediatric patients [18]. Symons et al. showed that CRRT is feasible and useful for children weighing 10 kg or less, and reported a similar survival rate in children who weighed 3 to 10 kg, as well as in older children [19]. In a recently published study on pediatric patients in our country, the mean age and patient weight at the time of CRRT were unrelated to survival [20]. CRRT is now considered for use in small infants, due to its increasingly widespread application and the growing experience of pediatric intensivists. Case reports and small series have described the use of CRRT in newborns [7,21–24]. Our study is one of the few studies that have reported the use of CVVHDF for inborn metabolic diseases during the neonatal period.

The optimal dose or preferred modality remains poorly defined. Continuous modalities may have advantages over intermittent HD as well as allowing safe electrolyte replacement and reducing ammonia rebound [21,25]. Jouvet et al. compared the three modalities (CVVHDF, CVVH, and CVVHD) in three infants with MSUD. They demonstrated that the removal of BCAAs with CVVHDF was slightly higher than with CVVHD, but markedly higher than with CVVH [26]. Picca et al. showed that HD has great efficiency in ammonia clearance, while CVVHD achieved the greatest clearance [27]. Schaefer et al. noted a 50% reduction in the ammonia level during a median 6.6 h with CVVHD [28]. In our study, all patients were supported with CVVHDF. The fastest ammonia extraction in a patient with UCD was a 44.8% reduction per hour. The reduction rate of metabolic toxins was 3.5%–44.8% per hour.

Reported complications related to continuous hemodialysis modalities are hemofilter clotting and associated blood loss [29]. Despite the use of heparin as an anticoagulant in the circuit, and regular dose adjustment, hemofilter clotting was the most common problem in our patients. Thrombocytopenia occurs in as much as 70% of patients receiving CRRT. Heparin-induced thrombocytopenia may occur, hemofilter consumption through the system besides the severity of illness of the patients, and the number of administered medications and comorbid conditions may contribute to the platelet loss during CRRT [30,31]. We observed thrombocytopenia in 62.5% of our patients, which thought to be occurred due to consumption through filter and system.

Inborn metabolic diseases are a particularly heterogeneous group of diseases that are responsible for significant morbidity and mortality. Neurotoxic metabolites that accumulate in neonates cause cell swelling and brain edema. The duration of exposure to such toxins associated with coma is directly correlated with the risk for neurological and developmental sequelae [32-34]. Mortality rates of 27.5% and 36% have been reported [35,36]. In our study, the mortality rate was 50%. In our country, the available human resources and services in centers of inborn metabolic diseases vary. Inborn metabolic diseases are common in eastern and southeastern regions of Turkey where the clinical infrastructure is insufficient, and metabolism specialists are less common in these regions than in others. On the other hand, carbamoylphosphate synthetase 1 (CPS1) deficiency for UCD or Mut0 mutation that results in no detectable methylmalonyl CoA mutase activity for MMA both have poor outcome, which may cause higher mortality rate in our study group. Therefore, almost all our patients who were unresponsive to medical and PD treatments had been transferred from different centers over considerable distances, which may have led to a longer exposure to toxic metabolites. Although we achieved a rapid decrease in metabolite levels, it did not reflect the mortality rate of the patients. 

Our series had some limitations. First, it was a single-center experience with a small study population. However, this is the first study from our country on the use of CVVHDF during the neonatal period for inborn metabolic diseases. Second, all patients were managed with the same CRRT modality, and thus we were unable to compare the effectiveness of different modalities. 

In conclusion, CVVHDF is a CRRT modality that can be used for the treatment of newborns with an inborn metabolic disease. Expeditious diagnosis is important, as is the prompt referral of an infant with a suspected or diagnosed inborn metabolic disease to a hospital with infant dialysis facilities. Early diagnosis and timely commencement of specific medical therapy, diet, and extracorporeal support, if needed, are likely to result in improved short and long-term outcomes.
